# *Drosophila* Gut—A Nexus Between Dietary Restriction and Lifespan

**DOI:** 10.3390/ijms19123810

**Published:** 2018-11-29

**Authors:** Ting Lian, Qi Wu, Brian A. Hodge, Kenneth A. Wilson, Guixiang Yu, Mingyao Yang

**Affiliations:** 1 Institute of Animal Genetics and Breeding, Sichuan Agricultural University, Chengdu 611130, China; lianting@sicau.edu.cn (T.L.); 18728153863@163.com (Q.W.); Yugx1102@163.com (G.Y.); 2 Buck Institute for Research on Aging, 8001 Redwood Blvd., Novato, CA 94947, USA; BHodge@buckinstitute.org (B.A.H.); kawilson@buckinstitute.org (K.A.W.); 3 Leonard Davis School of Gerontology, University of Southern California, Los Angeles, CA 90089, USA

**Keywords:** *Drosophila*, gut, aging, dietary restriction, intestinal epithelia barrier

## Abstract

Aging is often defined as the accumulation of damage at the molecular and cellular levels which, over time, results in marked physiological impairments throughout the organism. Dietary restriction (DR) has been recognized as one of the strongest lifespan extending therapies observed in a wide array of organisms. Recent studies aimed at elucidating how DR promotes healthy aging have demonstrated a vital role of the digestive tract in mediating the beneficial effects of DR. Here, we review how dietary restriction influences gut metabolic homeostasis and immune function. Our discussion is focused on studies of the *Drosophila* digestive tract, where we describe in detail the potential mechanisms in which DR enhances maintenance of the intestinal epithelial barrier, up-regulates lipid metabolic processes, and improves the ability of the gut to deal with damage or stress. We also examine evidence of a tissue-tissue crosstalk between gut and neighboring organs including brain and fat body. Taken together, we argue that the *Drosophila* gut plays a critical role in DR-mediated lifespan extension.

## 1. Introduction

Aging is often described as a lifelong process in which a variety of damages accumulate over time in molecules, cells, and tissues, thereby resulting in a decline in physiological function [1,2,3]. It is accompanied with a loss in proliferative homeostasis and regenerative capacity in high-turnover tissues, as stem cell pools become exhausted. This is the case in the ability of the gut to repair itself in old age [4,5,6]. Thus, the dysfunction of molecules, cells, as well as tissues, results in a range of aging-related diseases such as cancer and cardiovascular diseases [7,8]. The intestinal epithelium forms a selective barrier to allow nutrient absorption while keeping the microbiota within the lumen of the gut. To maintain proper gut homeostasis, the intestinal epithelia cells mount frequent and necessary immune responses against potentially harmful entities that are not maintained within the gut, such as pathogenic microorganisms, dietary antigens, and environmental toxins. Simultaneously, the intestinal epithelium is also involved in mutually-beneficial interactions with commensal life-forms that shape the host immune system, providing essential metabolic functions and permitting the absorption of nutrients, ions, and water [9,10]. In aging animals, the intestine suffers structural and functional impairments, thereby diminishing intestinal barrier function [11,12,13,14], which can promote other aging-related diseases such as cancer, inflammatory bowel disease (IBD), ulcerative colitis, and Crohn’s disease [15].

Dietary restriction (DR) has been demonstrated as one of the most robust interventions to extend lifespan across single-celled organisms, invertebrates, and vertebrate animals [16]. The term DR includes a broad range of interventions such as short-term starvation, periodic fasting, fasting-mimetic diets, intermittent fasting, normo-caloric diets with planned deficiencies (in particular macronutrients), and time-restricted feeding [17]. DR exerts its salutary effects by regulating evolutionarily conserved signaling pathways including major nutrient-sense pathways (insulin signaling and mTOR), stress-related pathways such as c-Jun N-terminal kinase (JNK) signaling, and pathways involved in intestinal proliferation such as JAK/STAT signaling [10,18,19,20]. Furthermore, the long-term maintenance of organismal homeostasis mediated by DR is dependent on interactions between organ systems [6,21]. Recently, the *Drosophila* intestine has emerged as an appealing model to explore tissue dynamics (i.e., regenerative capacity) with aging because of its genetic, morphological, and functional simplicity, and experimental accessibility by using sophisticated genetic tools as well as high structure similarity and evolutionary conservation of intestinal regeneration with humans [22,23]. In this review, we focus on how DR affects the *Drosophila* gut, and summarize the recent advances in our understanding of intestinal homeostasis throughout aging and its interaction in mediating benefits to lifespan and organismal health provided by DR.

## 2. Gut function During DR-Induced Longevity

### 2.1. Epithelial Homeostasis with Aging

**Figure 1:**
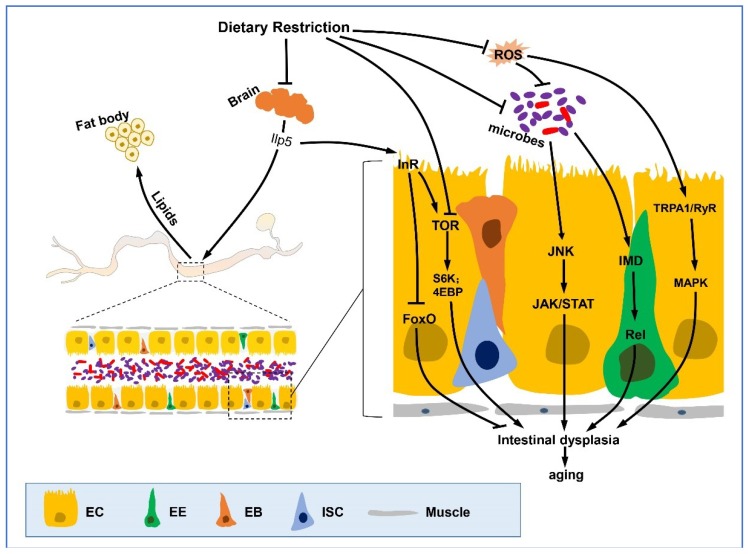
DR and DR mimetics improve gut epithelial function. In the fly gut, the epithelium consists of a monolayer of enterocytes (ECs) with interspersed enteroendocrine cells (EEs), and is basally located with intestinal stem cells (ISCs) and its daughter cells enteroblasts (EBs). The epithelial homeostasis of the gut is disrupted with aging, which causes dysplasia. Dietary restriction or its mimetics delay this process through various pathways including IIS signaling, TOR pathway, JNK, JAK/STAT pathway, IMD, and Ras/MAPK pathways. The communication of other organs with the intestine are also involved in the DR-mediated epithelial homeostasis and life extension effects. The secretion of Ilp5 from insulin-producing cells (IPCs) in the adult brain is inhibited by DR, which may signify a down-regulation of IIS, which then regulates TOR and FoxO indirectly. In addition, the fat body also mediates gut actions with aging by sharing lipid storage and metabolism with the intestine. Furthermore, DR reduces age-related intestinal flora growth rates, and down regulates JAK/STAT and IMD pathway, ultimately reducing ISC mis-differentiation and delaying functional degeneration of the intestine. Lines represent signaling pathways, arrows represent activation and blunt arrows represent inhibition.

The intestinal epithelium provides a selectively permeable barrier that functions to absorb nutrients while preventing the uptake of toxins and microbial contamination [24]. This barrier is maintained by self-renewing intestinal stem cells (ISCs) that sense damage and promote intestinal regeneration (Figure 1). ISCs constitute the majority of cells capable of mitosis in the *Drosophila* midgut epithelia, and respond to an array of different environmental stressors and nutritional conditions. ISCs thereby preserve the integrity of the intestinal barrier by adjusting epithelium size in response to changing stresses and dietary conditions [25,26]. In young flies or in states of low stress, ISCs are found to be in a ‘quiescent state,’ as their proliferation is relatively slow or non-existent [27], which makes it possible to replace the intestinal epithelium through symmetric division (one ISC divides into two ISC clones). This self-renewing division allows the stem cell pool to be scaled according to the needs of the gut tissue [26]. Throughout aging, environmental stress and damage result in accelerated ISC proliferation with asymmetric division, which is often referred to as the “proliferation state” [28]. ISCs generate daughter cells called enteroblasts (EBs). Unlike the mammalian intestinal crypts, in fly epithelium, ISCs reside in visceral muscle, while EBs localize apically to be mother stem cells. Ninety percent of EBs differentiate into polyploidy EBs to form the intestinal epithelium, and 10% appear to differentiate into either secretory enteroendocrine cells (EEs, small, diploid) or absorptive enterocytes (ECs, large, polyploid) [29,30,31,32,33]. Throughout aging, ISCs hyperproliferate and drive intestinal dysplasia [34]. In addition to intestinal dysplasia, a common hallmark of the aging gut is a progressive loss of barrier function, such that older guts lose the ability to selectively regulate nutrients and contain the microbiota in the intestinal lumen [4,12,35,36,37]. The intestines of elderly flies display an increase of stem cell proliferation, a loss of terminal differentiation of progenitor cells, increased intestinal flora, activation of inflammatory pathways, and increased intestinal permeability [37,38]. This loss of intestinal homeostasis is considered as a hallmark of aging in both flies and humans, and is associated with the progression of other aging-related diseases [39,40,41]. Intestinal epithelial barrier dysfunction has served as a predictor of mortality, as flies that have permeable guts display a decrease in longevity [12,35,42]. Our current understanding of the underlying molecular mechanisms that regulate intestinal epithelia maintenance and the age-associated loss of barrier function is limited, and is an active field of study.

#### 2.1.1. DR and DR Mimetics Improve Gut Epithelial Homeostasis

DR and treatment with DR mimetics such as rapamycin, 2,5-dimethyl-celecoxib (DMC), and metformin have been shown to promote gut epithelial homeostasis with aging [12,36,37,43]. In addition, consistent with DR mimetic pharmacological antiaging regimens, our recent work also shows that DR significantly reduces age-related intestinal flora growth rate (unpublished data). This suggests that lifespan extension by these therapies may be mediated in part by beneficial effects on gut health. Upon DR (and DR mimetics), flies display intestinal barrier loss at much slower rates compared to flies reared on control or nutrient rich diets. Rera et al. developed a noninvasive assay to determine individual fly intestinal integrity, and this assay has been used in many fly intestinal experiments [4,35,44]. In this assay, flies with loss of intestine barrier integrity are characterized by blue dye leaking from the intestinal tract into the rest of the body, and thus, are considered as “Smurf” flies. DR and DR mimetics significantly decrease the proportion of Smurfs in old age [12]. Secondly, intestinal size is diet dependent [26,45]. Flies fed a nutrient-rich diet show an increase of total intestinal cells due to the mis-differentiation of ISCs, which results in an increase in intestine gross size. The accumulation of ISCs and mis-differentiated daughter cells is significantly decreased in old flies on DR or DR mimetics. Among which, the number of esg- (transcription factor escargot, ISC- and EB-specific marker) and Delta- (Notch ligand, specifically expressed in ISCs) positive cells are significant decreased. Additionally, a decrease in the mitosis marker phosphorylated histone H3- (PH3) positive cells is also observed, which shows the decrease of ISC proliferation [4,37,44]. In addition, intercellular occluding junctions also show the critical role in maintaining intestinal barrier integrity such as tricellular junctions (TCJs), which is disrupted with aging. Giotactin (Gli) is localized to the TCJ in *Drosophila*. Renisk-Docampo et al. recently demonstrated that Gli is largely absent from the TCJ in old flies midguts, and depletion of Gli in ECs results in the impairment of intestinal regeneration, thereby accelerating loss of intestinal barrier integrity while DR delays the changes of Gli localization at TCJs in old flies [4,10]. Thus, DR shows the improvement of regenerative capacity by enhancing the expression of Gli at late age, and DR mimetics are able to maintain the regenerative capacity of intestinal stem cells population, which promotes flies with greater lifespan responses to DR.

#### 2.1.2. Pathways

Aging-related intestinal epithelial barrier dysfunction contributes to functional degeneration, including the disorder of intestinal immunity homeostasis from invertebrates to humans, as well as the incidence of cancer such as colorectal cancer [38,46]. ISC regeneration declining with aging has been shown to be regulated by both cell intrinsic and external environmental challenges [4,45]. Recent studies have demonstrated the involvement of a number of signaling pathways that regulate stem cell stress tolerance and repair. The precise coordination of protective and damage control mechanisms remain to be established. Here, we summarize the current signaling pathways that control ISC proliferation, differentiation, and the function in the context of DR-mediated longevity.

ISCs sense damage and proliferate throughout life, while with aging, they ultimately lose regenerative capacity, thus inducing an increase in ISC proliferation combined with the accumulation of mis-differentiated daughter cells [4,10]. DR and DR mimetics delay the over-proliferation of ISCs in old flies. The insulin (IIS) and target of rapamycin (TOR) nutrient signaling pathways communicate nutrient and energy levels to downstream transcriptional regulators that control ISC function (Figure 1).

DR and fasting reduce signaling through the IIS pathway, which is required for ISC proliferation and gut growth [26,47]. During fasting, low Insulin-like peptides (Ilps) result in ISC inactivity and smaller gut size. When food is abundant, diet ingestion acutely increases Ilp3 expression level in midgut visceral muscle and Ilp2 and 5 in brain. Elevated Ilp levels accelerate ISC proliferation rates, which then lead to increased cell number and promote gut growth [26]. In addition, protein restriction is also reported to decrease InR mRNA levels [48]. Genetic activation of the IIS pathway by expressing InR has been shown to induce intestinal dysplasia [27,49]. Limiting IIS signaling activity extends lifespan in flies [50,51]. Recent study reported that reducing IIS increases proteasomal assembly in *Drosophila* gut, and increases proteostasis, which maintains gut health [52]. Furthermore, reducing IIS also increased nutrient transport and storage in the gut via a FKH dependent manner [53], implying a potential role of enhanced intestinal heath in altered IIS induced longevity. In addition, transcription factor FoxO is repressed by IIS, and the activation of FoxO is required for the lifespan extension by reduced IIS in both *C. elegans* and *Drosophila* [54,55,56]. Though FoxO is not required for the longevity effect of DR, its activity modulates the response to DR in flies [57]. Loss of FoxO in mouse hematopoietic stem cells (HSCs) results in the increase of HSC proliferation and the elevation of reactive oxygen species (ROS) levels, consequently leading to the reduction of HSC pool regenerative ability [58,59,60]. However, if selected over-expression of the targets of FoxO such as *jafrac1* (a peroxiredoxin that detoxifies ROS) and *hsp68* (a heat shock protein) is able to limit the effects of IIS in the ISC lineage, this delays age-related intestinal epithelia dysfunction. In addition to FoxO-mediated cell-autonomous ISC proliferation mechanisms, IIS also nonautonomously regulates ISC proliferation; that is, InR is necessary to the EBs differentiation, and modest differentiation of EBs allows for further ISC division and then suppresses the aging phenotype-intestinal dysplasia in reduced IIS level flies [61]. These studies indicate that fly lifespan is extended when intestinal stem cell proliferation is reduced but not completely inhibited, and thus, highlight a key balance in promoting intestinal homeostasis. Likewise, age-related intestinal dysfunction is reported to be caused by the activation of the Jun-N-terminal Kinase (JNK) signaling pathway, which accelerates ISC activity and ultimately results in over-proliferation [34,38]. Reduction of JNK signaling activity in ISCs promotes lifespan extension in flies similar to reducing IIS signaling [27].

The nutrient responsive TOR signaling cascade has been widely demonstrated as a potent regulator of the aging process, as genetic or pharmacological inhibition of TOR have been shown to extend lifespan in a number of animal models [37,62,63,64]. Additionally, DR-mediated health benefits and longevity have been attributed in part to the decrease in TOR signaling activity. In fact, the lifespan extension effect of DR is blocked in TOR pathway mutant animals [65,66]. TOR, which is a serine/threonine protein kinase, integrates growth cues downstream of PI3K and AKT signaling cascades and regulates many downstream biological processes including mRNA translation, cellular growth, stress resistance, mitochondria biogenesis, autophagy, and stem cell function [67,68]. In flies, TOR is involved in maintaining stem cell identity and regulating differentiation of ISCs in a nutrient-dependent manner. TOR signaling plays a critical role for maintaining the stem cell pool by maintaining stem cell identity as well as ISC proliferation and promoting the symmetric differentiation of EBs into ECs and EEs. TOR activity is lower in ISCs than EBs since the TSC2 protein, which acts as a suppressor of TOR, is highly expressed in ISCs but not in EBs. Inhibition of TSC2 downstream of the Notch signaling pathway in EBs activates TOR, and thus promotes the commitment of EBs into the EC fate [47,69]. These observations are consistent with studies in mice that demonstrate lower activity of mTOR is detected in Paneth cells (which is the ISC-supporting cells) under DR, which regulates ISC regeneration through mTOR by sensing the organismal nutritional status [70]. Recent reports show, however, that mTOR activity is up-regulated in ISCs upon DR, which forces ISC proliferation. Rapamycin treatment, which represses TOR activity, acts as a DR mimetic by blocking ISC expansion in mice fed in DR conditions with suitable doses [71]. Thus, drugs like DR mimetics such as rapamycin may cause opposite effects on different cell types. The concise molecular regulating mechanisms of TOR in ISC proliferation and ISC lineage differentiation need to be explored further.

The intestinal epithelium is continually challenged by pathogenic bacteria, as well as the commensal microbiota which can influence intestinal homeostasis, immune stress responses, and the regenerative activity of the epithelial tissue. To combat potentially harmful pathogens, the intestinal epithelium will respond to damage by increasing the expression of antimicrobial peptides (AMPs). AMPs are mainly regulated by the Toll and Immune Deficiency (IMD) innate immune pathways [72,73]. In addition, AMPs can be directly activated by the transcription factors *Drosophila* Forkhead boxO (dFoxO) or Forkhead (FKH). In consideration of the fact that FoxO and FKH are directly repressed by IIS and TOR signaling [74], this could suggest a potential mechanism that DR may increase AMPs expression in a IIS- and TOR-dependent manner [37,44]. In the fly midgut, AMPs are regulated by IMD, the Janus kinase-signal transducers and activators of transcription (JAK-STAT) pathways [75], and caudal, a negative transcriptional regulator [76], but not Toll signaling. Ubiquitous or gut-specific over-expression of *Dro* (one of AMPs–*Drosocin*), increases *Drosophila* lifespan and is accompanied by the reduction of AMPs in the fly midgut, as well as JNK Epidermal growth factor receptor (EGFR), which is required for intestinal regeneration and ISC pool homeostasis [77]. These pathways are usually regarded as the makers of intestinal homeostasis because of their elevated activity with aging or response to bacterial challenge [78,79]. Changes in the composition of the microbiota can trigger chronic JNK and JAK-STAT signaling activity with aging, which in turn, promotes ISC over-proliferation, resulting in intestinal epithelia dysplasia [38]. Furthermore, Loch and colleagues also observed that gut permeability is significantly decreased in *Dro* over-expressing flies with aging. This improved intestinal barrier is also observed in DR flies, eliciting the crosstalk of nutrient, innate immunity, intestinal homeostasis, and aging [77].

### 2.2. Intestinal Lipid Homeostasis

Maintaining proper lipid metabolic homeostasis is central to organismal health. Disrupting lipid synthesis and/or breakdown is a major risk factor for metabolic diseases such as obesity, type-2 diabetes, and cardiovascular diseases [80,81]. Under normal conditions, lipid homeostasis is maintained by absorption of dietary lipids through the intestinal epithelium into the circulation where peripheral tissues can either store excess lipids or metabolize them for energy. The process of dietary lipid absorption begins with the breakdown of lipids (including triacylglycerol (TAG) and cholesterol esters) into free-fatty acids (FFAs), monoacylglycerols, and free sterols in the intestinal lumen [82,83]. FFAs are absorbed by ECs in the intestine and are resynthesized into TAGs, and then packaged into lipoprotein particles together with cholesterol, cholesterol esters, and carrier proteins [19]. These lipoprotein particles are trafficked to peripheral tissues. These lipids can be either used by cells for energy or deposited in storage tissues such as the fat body and intestine [81]. Thus, as stated above, the misdifferentiation of EBs to ECs with aging will disrupt the lipid metabolism in intestine, thereby influencing organismal health.

#### 2.2.1. DR Maintains Intestinal Lipid Homeostasis

**Figure 2:**
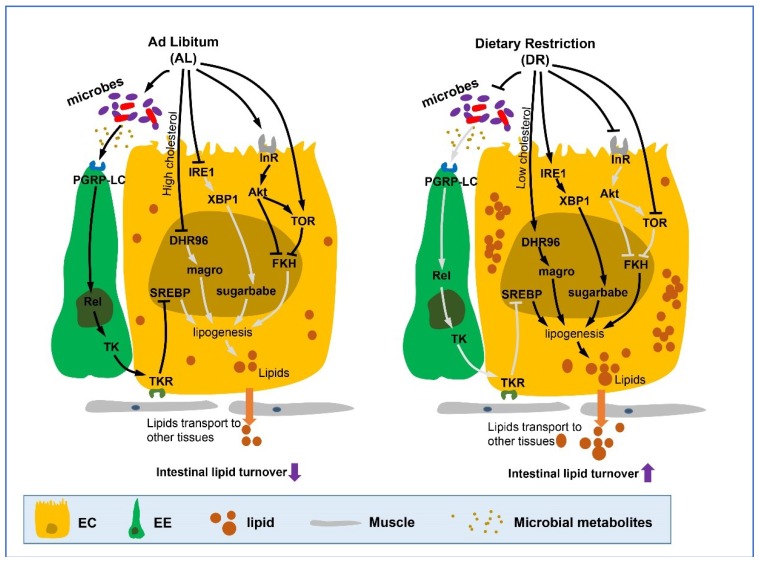
DR up-regulates intestinal lipid metabolism. Dietary restriction or its mimetics promote the adaptation towards triglyceride usage, increase lipid accumulation and fat storage in the fly gut, and also increase lipid transport to other tissues from the gut. This process is mediated by a range of hormones and pathways including Akt/TOR pathway, microbes regulated IMD pathway, endoplasmic reticulum (ER) stress related IRE1/XBP1 signaling module which ultimately induce the expression of lipid metabolism-related genes or transcription factors such as magro, sugarbabe, and FKH in nucleus. It should be noted that nuclear receptor DHR96 is activated in low cholesterol dietary conditions (cholesterol restriction) which then causes an increase in the transcription of the downstream bifunctional enzyme magro (with both Gastric lipase and cholesterol lipase activity), increasing the digestion of dietary TAG and cholesterol esters in lumen and lipogenesis in ECs. (Lines represent signaling pathways, arrows represent activation and blunt arrows represent inhibition. Light gray lines refer to path which is blocked or inhibited). EC, enterocyte; EE, enteroendocrine cell.

Studies have characterized the molecular mechanisms of lipid uptake, synthesis, catabolism, and mobilization taking place in the intestine [19,84,85,86,87] (Figure 2).

In the fly, there is a progressive loss in the intestine’s ability to synthesize and store lipids with aging because of the decline in the number of ECs in the intestine, resulting in decreased ability to transport lipids or absorb lipids from the lumen into ECs. Restoration of intestinal lipid metabolism has been reported to extend lifespan in flies [88]. DR improves intestinal epithelia barrier function and also promotes a metabolic shift towards enhanced utilization of lipids and increased mitochondrial function [86,89,90,91]. DR promotes the conversion of dietary carbohydrates into lipids, increases the synthesis and breakdown of fatty-acid, and accelerates lipid turnover in flies. Knockdown of the TAG synthesis gene *Acc* ablates DR-mediated lifespan extension, which highlights the importance of lipid metabolism upon DR [86,92].

Inhibition of IIS and TOR is well-known to affect fat metabolism in *Drosophila* [50,93]. A recent study suggests that FKH promotes intestinal lipid storage in response to reduced IIS and may also mediate the enhanced lipids in TOR inactivation [53]. Another pathway known to be involved in lipid metabolism in *Drosophila* gut is a steroid hormone pathway that includes DHR96, a nuclear hormone receptor (NHR) related to cholesterol metabolism. DHR96 nuclear receptor was reported to bind cholesterol and regulate cholesterol and TAG homeostasis. In cholesterol restriction conditions, DHR96 was activated, which then increased the transcription of a direct target, magro (a bifunctional enzyme, which has both TAG lipase and cholesterol esterase activities), in the anterior gut. This resulted in increasing the catabolism of dietary TAG and cholesterol esters in lumen and lipogenesis in ECs [87,94,95,96].

Tachykinin (TK) is a prohormone in midgut EEs [97]. TK encodes 6 mature peptides (TK1-6), which are expressed in the anterior, middle, and posterior midgut [98]. Gut prohormones promote gut contraction and maintenance of gut peristalsis [99,100]. Song and colleagues revealed the physiological role of TK in regulating intestinal lipid homeostasis. This group showed that TK represses lipogenesis in ECs through TKR99D (a G-protein-coupled TK receptor in the gut) and protein kinase A (PKA) signaling [84,101,102]. Recently, a study revealed that TK expression can be regulated by the microbial metabolite acetate through the IMD pathway, which induces a reprogramming of lipid metabolism [103]. In consideration of DR forcefully decreasing gut microbes (our recent unpublished data), it can be suggested that DR increases lipid metabolism in a TK-dependent manner.

Moreover, endoplasmic reticulum (ER) stress has been shown to link lipid homeostasis and human diseases, including diabetes and metabolic syndrome [104,105]. During ER stress, the transducer IRE1 regulates ER homeostasis by inducing the activity of genes involved in ER biogenesis and protein folding and degradation through dimerizing and splicing XBP1 [106]. Both IRE1 and XBP1 are required for lipid homeostasis with increased lipogenesis and lipid usage [107,108,109,110]. Recently, the novel role of the IRE1/XBP1 ER stress signaling module in ECs was established, showing that it regulates the shift towards the increase of intestinal TAG usage upon DR associated with sugarbabe (a Gli-like zinc-finger transcription factor) [19], which is consequently beneficial for lifespan. This suggests that IRE1/XBP1/Sugarbabe signaling mediates the metabolic adaptation of intestinal epithelium upon DR.

### 2.3. DR Improves the Intestinal Oxidative Stress Resistance

DR has been reported as the anti-aging paradigm in protecting against oxidative stress-induced diseases through reducing reactive oxygen species production, increasing antioxidant enzyme activity, as well as increasing the turnover of oxidized macromolecules [111]. Fly intestine is thought to be a simple model to characterize the increased ability of oxidative stress induced by aging or oxidants such as paraquat and H_2_O_2_ [36,112,113,114].

Oxidative stress increases with aging and age-related diseases such as cancer, neurodegeneration, cardiovascular disease, and diabetes [111]. Oxidative stress is caused by an imbalance in the rate of reactive oxygen species (ROS) production and detoxification [115]. Higher ROS levels are observed with tissue damage and aging, or induced by the administration of exogenous oxidants such as paraquat and/or hydrogen peroxide in flies midgut [116,117,118]. Oxidative stress can damage intracellular macromolecules, which results in the disruption of protein/gene expression, cellular dysfunction, and death. Over time, accumulating damage caused by ROS can accelerate aging and age-related diseases [119,120,121].

In *Drosophila*, mitochondria are the major generators of ROS, and complex I is the main source of ROS associated with aging. Damaged mitochondria usually produce excess ROS. In long-lived organisms, there are lower ROS levels produced at complex I, and the inhibition of electron transport chain (ETC) complex can act like a DR mimetic [122]. Thus, ROS is an attractive candidate for targeting the aging process. Damaged mitochondria can be degraded by mitophagy (mitochondria-targeted selective autophagy), which is regulated by PINK1/Parkin pathway. Increased levels of autophagosomes by DR and DR mimetics promote the elimination of damaged mitochondria-induced mtDNA oxidative damage, mitochondria free radicals, and ROS [36,37,123,124]. In addition, DR can activate the expression of dPGC-1 (*Drosophila* PGC-1 homolog, peroxisome proliferator-activated receptor-γ coactivators, from our unpublished RNA-seq data), which plays a key role in mitochondrial biogenesis and respiration [125,126] in *Drosophila* and mammals. Overexpression of dPGC-1 is sufficient to increase the activity of mitochondria in the intestinal epithelium, lower ROS level, and delay the accumulation of misdifferentiated ISCs, thereby improving gut homeostasis and extending lifespan [35]. One recent report demonstrated that when ISCs sense oxidative stress, TRPA1 and RyR are identified to regulate cytosolic Ca^2+^ level in ISCs to activate (by src) and amplify (via autocrine Spi-EGFR signaling) the downstream EGFR-Ras/MAPK signaling, thereby inducing ISC proliferation [121]. p38 MAPK signaling has been reported to maintain fly intestinal host defense and metabolic homeostasis, especially p38c. In the guts of p38c fly mutants, ROS levels are significantly decreased upon bacterial infection [114]. Thus, DR might extend fly lifespans through decreasing mitochondrial free radicals, which is regulated by mitophagy, as well as improving the mitochondrial respiration chain activity.

### 2.4. How Gut-Other Organs Communication Contributes to the Benefits of DR

DR-mediated intestinal homeostasis is maintained through a range of signals that originate within the intestine but also through autocrine/paracrine signaling from neighboring tissues. So far, a number of previous studies suggest that the communication of the gastrointestinal tract (GI tract) and the neighboring tissues is contributable to maintain the homeostasis of DR benefits which occur in the intestine [6,21]. Here, we briefly review the communication signals between the GI tract and neighboring organs, including brain and fat body, upon DR.

#### 2.4.1. Gut-Brain

As stated above, IIS is not required for life extension of DR, but its activity modulates DR response [57] (also see review [23,127]). Insulin-like peptides (Ilps), which regulate the activity of IIS, are primarily secreted from insulin producing cells (IPCs) (median neurosecretory cells, MNCs) in the adult brain, including Ilp1, 2, 3, and 5 [128]. Ilp 5 is also produced in adult ovarian follicles and renal tubules, while dilp3 is expressed in the midgut. Other Ilps are expressed in larval fat body, embryonic mesoderm, and adult central nervous system among other tissues [48]. Only Ilp2, 3, and 5 are likely to mediate the response of diet, since both Ilp5 mRNA and protein levels are down-regulated in *Drosophila* brain upon a yeast-diluted DR diet, and fasting also reduces Ilp2 in brain, potentially implying crosstalk between the brain and gut under DR conditions [26,129,130]. However, it should be noted that losing Ilp5 does not diminish the capacity of DR to extend lifespan [130]. In addition to the direct influences on Ilps production, other interesting gut-secreted signaling is also documented. For example, AMPK activation in the intestine can regulate autophagy in the brain through signals such as the activity of *Autophagy-specific gene 1* (*Atg1*), and *Atg1* in turn maintains intestine homeostasis [131]. AMPK is also activated under DR and DR mimetics in flies [132]. Taken together, the communication between the brain and gut through the regulation of Ilp production and other metabolisms in response to DR consequently have an effect on lifespan.

#### 2.4.2. Gut-Fat Body

A number of previous studies have shown the communications of gut and fat body in regulating systemic homeostasis including lipid metabolism, AMP production, and the role of the fat body in mediating intestinal actions [6,19,84]. Firstly, gut shares lipid storage and metabolism with the fat body [84]. Loss of TK in fly EEs increases lipid levels in the fat body, and excesses of gut TK levels with deprivation of food induces the loss of systemic lipid storage in fat body through inhibiting sterol regulatory element-binding protein (SREBP) [84]. In addition, high levels of neurotensin (NT) in the EEs increase the lipid accumulation in the fat body with decreasing AMPK activation [133]. Secondly, the gut and fat body are responsible for controlling and regulating systemic AMP production. AMPs are secreted by the fat body, which is modulated by gut-expressed PGRP-LE. PGRP-LE can be repressed by gut-expressed amidase peptidoglycan-recognition proteins (PGRPs) including PGRP-LB and PGRP-SCs [134,135]. Fat body signaling also mediates gut actions with aging. Specific loss of lamin-B in the fat body results in the loss of intestinal epithelium regeneration mediated by the IMD pathway [136]. Additionally, nutrient-signaling pathways, such as transforming growth factor β (TGF-β) signaling, are also involved in intestinal ingestion and absorption. Fat body-secreted Dawdle (Dw), the TGF-β ligand, is responsible for the regulation of carbohydrase and lipase levels within the midgut through Smad2 [137]. TGF-β levels are regulated in different adipose tissues in mice upon energy restriction, but the question of whether DR regulates this pathway or not needs to be investigated further [138]. As we discussed above, AMPK and IMD are up-regulated under DR and DR mimetics [37,132], which suggests the regulation of DR benefits in fatty acid accumulation and the improvement in systemic homeostasis.

## 3. Conclusion

Studies in the simplified *Drosophila* intestine have yielded significant research progress in demonstrating the molecular mechanisms of nutrient response to an organisms’ lifespan. Here, we have summarized the present knowledge regarding the role of DR in promoting homeostasis of the intestine epithelial barrier, lipid metabolism, and stress responses. Furthermore, the communication between the intestine and the neighbor tissues is briefly discussed, suggesting organ-organ crosstalk may play a role in promoting the beneficial effects of DR on the gut. While we are beginning to unravel the molecular mechanisms that control the different cell populations in the gut, the question of how DR affects these individual processes with age remains to be studied. The *Drosophila* and mammalian intestine are sharing many similarities at the molecular and cellular levels [28,38,139]. Therefore, a more comprehensive understanding of *Drosophila* intestinal physiology and pathology in response to aging and different dietary interventions may translate into findings in higher order animals and humans.

## Abbreviations

AMPK	AMP-activated protein kinase
AMPs	Antimicrobial peptides
Atg1	Autophagy-specific gene 1
CCK	Cholecystokinin
DMC	2,5-dimethyl-celecoxib
DR	Dietary restriction
EB	Enteroblast
EC	Enterocyte
EE	Enteroendocrine cell
EGFR	Epidermal growth factor receptor
ER	Endoplasmic reticulum
ETC	Electron transport chain
FFA	Free-fatty acid
Fkh	Forkhead
FOXO	Forkhead boxO
GLP	Glucagon-like peptide
HSC	Hematopoietic stem cell
IBD	Inflammatory bowel disease
IIS	Insulin/IGF-1 signaling pathway
Ilps	Insulin-like peptides
IMD	Immune Deficiency
InR	Insulin/IGF-1 like tyrosine kinase receptor
IPC	Insulin producing cell
ISC	Intestinal stem cell
JNK	Jun N-terminal kinase
mTOR	Mechanistic target of rapamycin
PGRP	Peptidoglycan-recognition protein
PI3K	Phosphatidylinositol 3-kinase
PKA	Protein kinase A
ROS	Reactive oxygen species
SREBP	Sterol regulatory element-binding protein
TAG	Triacylglycerol
TCJ	Tricellular junction
TGF-β	Transforming growth factor β
TK	Tachykinin
TSC2	Tuberous sclerosis complex protein 2
